# Health Information Needs of Breast Cancer Survivors: An Umbrella Review

**DOI:** 10.1155/2024/5889622

**Published:** 2024-07-15

**Authors:** Nahid Gavili, Shahram Sedghi, Sirous Panahi, Maryam Razmgir

**Affiliations:** Department of Medical Library and Information Science School of Health Management and Information Sciences Iran University of Medical Sciences, Tehran, Iran

## Abstract

**Purpose:**

The aim of this umbrella review was to identify the main information needs of breast cancer survivors. Since several reviews have already been done on this topic, conducting an umbrella review not only combines their results but also gives a comprehensive picture and informative summary of breast cancer survivors' needs.

**Method:**

The search was performed in PubMed, Embase, Scopus, Web of Science, ProQuest, Cochrane, and Google Scholar from inception to the end of March 2024. This review was conducted according to the JBI methodology for umbrella reviews, and the report was based on Rutten's category for information needs of patients with cancer. After removing duplicate and irrelevant articles, 14 systematic reviews were included in the analysis. The JBI checklist was used for evaluating the quality of eligible articles.

**Results:**

The information needs were classified into 11 main categories and 86 subcategories. As a result of this umbrella review, one category was added to Rutten's 10 categories. Also, treatment information needs were introduced as the main identified category. Information on supportive care needs ranked second, and body image/sexuality information needs ranked third with a slight difference.

**Conclusion:**

The information needs outlined in the present study can serve as a general model to help clinical decision makers and policymakers in order to better understand the needs of the group and meet the information needs of the population. *Implications for Cancer Survivors*. These recommendations can promote and develop targeted interventions to reduce the psychosocial consequences of breast cancer survivors and increase their quality of life.

## 1. Introduction

Information needs have been a topic of discussion in the 21st century, largely due to the impact of globalization trends and the growth of the information society [1]. The information needs imply the inadequacy of one's knowledge to achieve certain goals [2]. In the healthcare setting, health information needs refer to the lack or inadequacy of information about a patient's lifetime [3]. As for a life-threatening disease such as cancer, and due to its complex treatment, patients experience long-term suffering from clinical, emotional, and social aspects and, consequently, require a wide range of information to meet relevant needs [4, 5].

Cancer is a noncommunicable disease and one of the most common causes of death in the world [6]. It is also considered one of the significant challenges for modern healthcare systems [7]. According to the latest estimation of the International Agency for Research on Cancer (IARC), women's breast cancer is the most common cancer diagnosis worldwide, having surpassed lung cancer for the first time in 2020. By 2040, the incidence of breast cancer is expected to rise to 3 million new cases per year [8].

In recent years, parallel to the rising prevalence of breast cancer worldwide, the patient's survival rate has increased thanks to advanced medical technology and biology (20), organized screening plans [9], progress in early diagnosis [10], and the rise of combination therapies such as chemotherapy, hormone therapy, and radiotherapy [11]. Breast cancer survivors constitute the largest group of cancer survivors with particular needs [12, 13]. Because the term breast cancer survivors includes different definitions, in the present study, following the American Cancer Society definitions, the term “breast cancer survivor” was used to refer to any individual with a definite diagnosis of breast cancer, irrespective of the disease stage [14]. Also, this review focuses on the sexual, psychological, and supportive care needs of breast cancer survivors. Supportive care is an individualized approach to providing services to people affected by cancer so as to fulfill their informational, emotional, spiritual, social, and physical needs during diagnosis, treatment, and follow-up stages [15].

Cancer does not solely affect the patient's personal life [14]; rather, it imposes a heavy burden on the healthcare system, patient's family, and whole society [16]. Consequently, the patient, the family, healthcare providers, as well as the entire society encounter clinical, emotional, and social challenges [5, 17]. Thus, recognizing patients' experiences and identifying health information needs of this group not only facilitate understanding risk circumstances and critical periods but also help stakeholders take appropriate measures to respond to challenges (30), enhance chances of survival, and empower women to actively deal with cancer [18].

In their systematic review, Rutten et al. classified the information needs of patients with cancer into 10 categories: cancer-specific information, treatment-related information, prognosis information, rehabilitation information, surveillance and health information, coping information, interpersonal/social information, financial/legal information, medical system information, and body image/sexuality information [19]. Although other systematic reviews have proposed various dimensions of information needs in breast cancer survivors, including supportive care [20], persistent pain after treatment [21], psychosocial considerations [22], mindfulness interventions [23], and chemotherapy experiences [24], no comprehensive study has attempted to summarize and synthesize relevant findings. Even an umbrella review and a meta-review published in 2023 and 2024, respectively, addressed the unmet supportive care needs in several cancers and the psychological needs of women with breast cancer [25, 26].

Therefore, conducting the present research seemed essential from two perspectives. First, breast cancer has turned from a fatal disease into a chronic disease thanks to the rising population of cancer survivors and advances in cancer therapies. Second, female survivors of breast cancer, as a distinct group of society, undergo psychological disorders such as depression, anxiety, embarrassment, feelings of unattractiveness, and sexual dysfunction due to changes in their appearance and body image.

The chronic nature of breast cancer and the sensitivity of the target population have caused various challenges for breast cancer survivors compared to the survivors of other types of cancer; therefore, the information needs of this group seems to differ from the majority of other cancer survivors. Therefore, the present study was conducted using the umbrella review method, which is a novel approach to summarizing and synthesizing current scientific findings [27]. In addition to providing a detailed report of systematic reviews about the health information needs of breast cancer survivors, the present umbrella review attempted to identify the specific needs of this population and provide evidence to clinical decision makers for promoting and developing targeted interventions to reduce the psychosocial consequences of breast cancer survivors and increase their quality of life. It is our hope that the findings help to shape a comprehensive road map for researchers, clinical decision-makers, and policymakers.

## 2. Methods

### 2.1. Objectives and Research Questions

This umbrella review was conducted according to the JBI (Joanna Briggs Institute) guidelines. As a relatively novel methodology, an umbrella review intends to systematically search, evaluate, and organize multiple systematic reviews (with or without meta-analysis) about identified outcomes associated with a certain exposure [28]. The main purpose of these studies is to summarize and synthesize a comprehensive picture of concepts and existing evidence, by visualization in the form of charts and tables [29]. Umbrella reviews also provide a combination of the following two levels of evidence: systematic reviews and their own original investigations [28]. In fact, systematic reviews are units of analysis in umbrella reviews. From this perspective, umbrella reviews can be regarded as the secondary study of secondary studies or a review of systematic reviews, featuring the highest levels of summarizing evidence and synthesizing biomedical literature [29].

This umbrella review sought to answer the following question: “what are the health information needs of women surviving breast cancer?”

### 2.2. Search Strategy

Systematic reviews in PubMed, Embase, Scopus, Web of Science, ProQuest, and Cochrane database were the focus of our searches. To retrieve the grey and nonindexed literature in the mentioned databases, Google Scholar was searched as an academic search engine. The keywords “information need,” “breast cancer,” and “women” shaped the key components of our searches. In order to cover both controlled and free keywords, we looked up related and synonymous keywords in Medical Subject Headings (MeSH), consulted subject experts, and extracted keywords from related and highly cited articles. The search strategy was implemented in the selected databases without time restriction, by applying the limiter of the “type of article” (review, systematic review, and meta-analysis) if it ever existed in a given database. To increase the recall percentage in the retrieval process, we only applied the limiter of “review articles,” and identified systematic reviews during the screening process. Also, to ensure the maximum retrieval of potentially relevant studies, we performed backward searching (chain searching) within the references of included studies. Web of Science, Scopus, and Google Scholar were used for forward citation searching to find the related results as far as possible. The strategies used in mentioned databases and search engines are given in Table 1 in supplements.

### 2.3. Inclusion and Exclusion Criteria

The most important inclusion and exclusion criteria of the articles were as follows (see Table 2).

### 2.4. Study Screening and Selection

**Figure 1:**
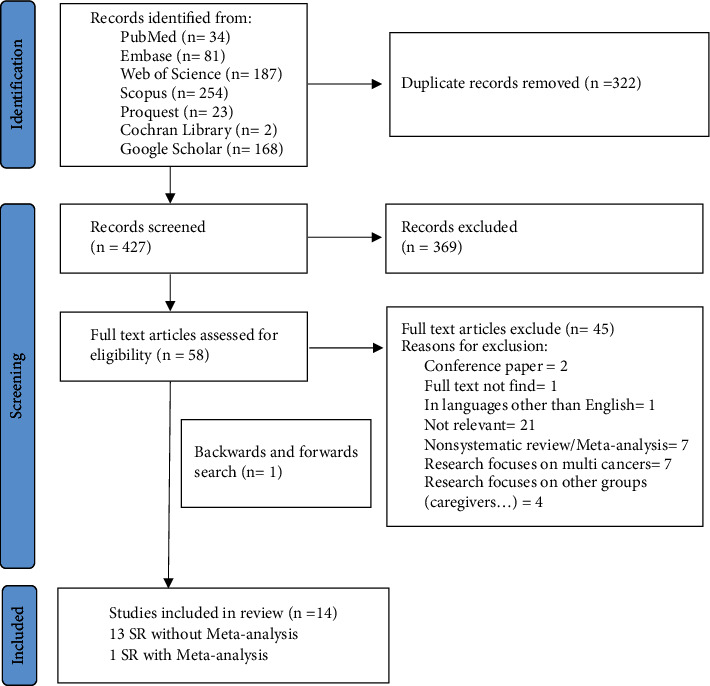
Study selection process using the PRISMA flow diagram.

The search was performed between 20 and 29 March 2024 within the mentioned databases. Totally, 749 documents were retrieved. All results were exported to Endnote X10. After removing the duplicate documents (*n* = 322), 427 documents were obtained, and their titles and abstracts were screened independently by two contributors. Also, 369 out of 427 articles were eliminated due to irrelevance or incompatibility of their objectives with the present study; finally, 58 articles remained to be thoroughly reviewed. After reviewing the articles' full text, we excluded 45 other articles for other reasons listed in Figure 1. In addition, the references of the retrieved studies were manually searched for additional publications and one further relevant study was also identified, resulting in a final total of 14 included articles. All included studies that met the objectives of the present study were ensured to undergo in-depth analysis, categorization, and summarization of the evidence. The bibliographic and thematic data of these articles were collected using the checklist recommended by the JBI guidelines for systematic reviews.

Data extraction was independently conducted by 2 contributors (N.G and M.R). In case of disagreement over extracting the details of the eligible studies, we sought the opinion of a third contributor. In the next step, each article was analyzed as an independent unit. In order to summarize the concepts and to categorize the articles, we used MAXQDA (2020) as a qualitative data analysis tool.

### 2.5. Quality Assessment

The Critical Appraisal Checklist for Systematic Reviews and Research Syntheses was used in order to ensure the quality of included studies and reduce bias [28]. The review process was done independently by 2 contributors (N.G and M.R) from the team of authors. Any disagreements were resolved by discussion with a (second) author. According to the decision of the researchers, 6 out of 11 were considered as the cutoff point; any of the evaluated studies scoring less than 6 were excluded from the present study. But the qualitative assessment showed that none of the articles received a score less than 6; therefore, all 14 articles were found eligible for further analysis. The results of quality assessment are detailed in Table 3.

### 2.6. Data Extraction

The key data of the studies that met the inclusion criteria were independently extracted by two researchers of the present study (N.G and M.R) (displayed in Table 4 in supplements). Following the JBI checklist, we extracted the data of each qualified systematic review within the study details (authors, year of publication, objectives, and participants), search details (database sources, range of included studies, number of studies included, types of studies, and their country of origin), and analysis (main findings/results).

Overall, 13 systematic reviews without meta-analysis and 1 systematic review with meta-analysis were included. Due to the insufficient data of the systematic review with meta-analysis in providing a detailed quantitative report, we merely assessed the descriptive information of that meta-analysis and did not provide a separate quantitative report.

## 3. Results

**Figure 2:**
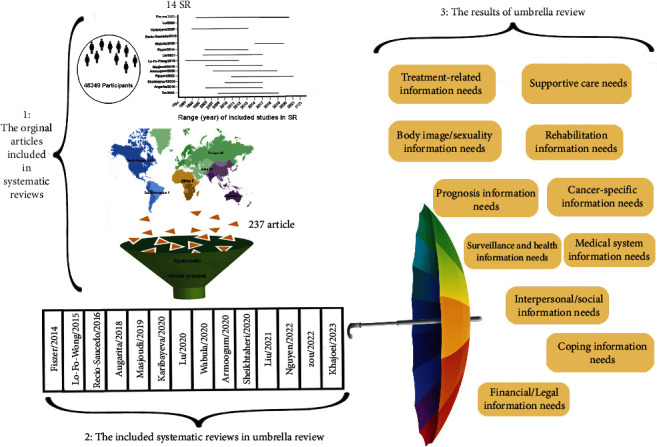
Visual representation of the present umbrella review health information needs of breast cancer survivors.

Two components, namely, systematic reviews and their primary studies, play a crucial role in the results of an umbrella review. Therefore, a descriptive summary of primary studies is presented in the first part of Figure 2. Also, to facilitate visualization of key evidence, the 14 included systematic reviews and the summarised categories of health information needs of breast cancer survivors have been illustrated in the second and third sections.

The data of section one show that 48,349 people participated as samples in 237 original articles between 1994 and 2021. Geographically, these studies were conducted (The setting of original papers was not available in some studies) in Asia (*n* = 78), Europe (*n* = 54), North America (*n* = 63), Australia (*n* = 26), Africa (*n* = 1), and South America (*n* = 1). The distribution diagram showing the publication years of the original articles indicates that most of the articles addressing the information needs of women with breast cancer were published between 2000 and 2017.

The second section of the illustrated synopsis deals with the 14 systematic studies included in the current research, which were conducted between 2014 and 2023 in China (*n* = 3), England (*n* = 2), Iran (*n* = 3), Canada (*n* = 1), Vietnam (*n* = 1), France (*n* = 1), Indonesia (*n* = 1), Kazakhstan (*n* = 1), and the Netherlands (*n* = 1). These systematic reviews were observed as the output of systematic review process at the first level, as well as the principal input of the umbrella review at the second level. The third section of the illustrated synopsis concerns' results of the present umbrella review and shows the 11 main categories of health information needs of breast cancer survivors, which is the main finding of the present study.

Table 5 summarizes the findings of the current study in 11 main categories and 86 subcategories of information needs. According to the combined evidence and to answer the main question of the review, one further category (supportive care needs) was added to the 10 main categories previously reported by Rutten et al. Also, 50 new subcategories were added, and 11 subcategories were proposed to be either merged (while keeping the previous title), expanded, replaced, or renamed. It is important to note that a total of 64 subcategories have been introduced by Rutten et al., whereas in this umbrella review, only those subcategories were used that were mentioned as needs in 14 included systematic reviews.

**Figure 3:**
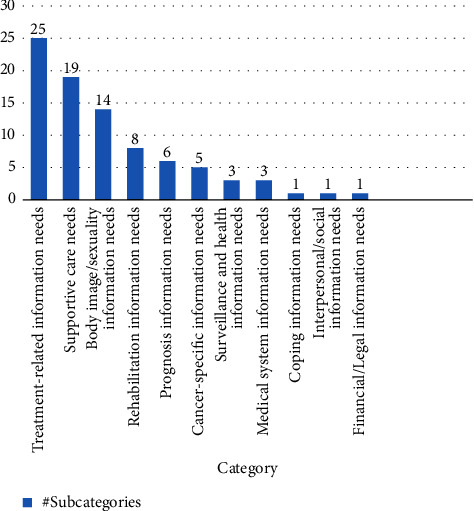
Frequency of subcategories.

The present umbrella review identified different information needs at different stages of participants' experiences of cancer.  Figure 3 displays the frequency of subcategories of information needs in each major category.

In the following sections, we will introduce the main categories as well as some of the more frequent subcategories.

### 3.1. Treatment-Related Information Needs

In the present review, information needs related to the treatment category were found (in 25 subcategories) to be more frequent than other information needs. Also, treatment-related information needs were reported across 10 out of 14 included systematic reviews. Similarly, Sheikhtaheri et al. reported that information needs on both treatment and diagnosis jointly constituted a top priority among the information needs of breast cancer survivors [34].

Based on our findings, the subcategory of “available treatments/treatment options” had the highest frequency among the subcategories of treatment-related information needs. The results further showed that it was one of the common information needs of breast cancer survivors, especially among older women with non-metastasized breast cancer as well as younger women undergoing treatment with mastectomy or breast-conserving therapy [23, 34, 38]. However, the findings reported by Amiri et al. were slightly different from ours, suggesting that despite patients' need for information about future treatment options, they were already familiar with therapeutic options introduced by physicians, and this need had been met over time [6]. In addition, the summarized evidence showed that patients had a strong need for information about “side effects of treatment/risks and benefits of treatment/management of side effects” [34]; this need becomes even more conspicuous in young women surviving breast cancer [38]. In this regard, Papadakos et al. reported that the most important information needs of patients with cancer were related to prevention, treatment, and side-effects' management, as well as information on possible complications caused by treatment methods [41]. Riccetti et al. reported information needs related to the side effects of treatment methods among Chinese migrant cancer patients, as well as Native American, Native Alaskan, and Native Hawaiian cancer survivors [42].

### 3.2. Supportive Care Needs (Information Needs and Features of Information, Spiritual Needs, Social Needs, Physical Needs, and Emotional Needs)

The results of this review further revealed that supportive care was a basic requirement of breast cancer survivors. Accordingly, these needs were identified in 19 subcategories, with the highest frequency in “information needs and the features of information,” “physical needs,” and “social needs” [21, 24, 32, 38, 40]. Besides, Riccetti et al. observed in their umbrella review that Native American, Native Hawaiian, and Alaskan cancer survivors frequently expressed the need for the participation of their family members and community in supportive care programs. Moreover, the need to incorporate spiritual activities and take educational measures to remove the cancer stigma was emphasized in the support groups [42].

In an umbrella review of supportive care needs of various cancer survivors, Paterson et al. found that the most unmet supportive care needs were as follows in the descending order: psychological/emotional, health system/information, interpersonal/intimacy, social, physical, family, practical, daily living, spiritual, patient-clinician communication, and cognitive [26].

### 3.3. Body Image/Sexuality Information Needs

According to the findings of this review, the information needs related to body image and sexuality were reported by breast cancer survivors in 14 subcategories. An eligible study reviewed in the present umbrella review reported that this sort of information about cancer patients' acceptance of disease and body image and sexual health ranked third and fifth among information needs, respectively [32]. Riccetti et al. also confirmed that information needs about body image and sexuality were frequently reported among Latino/Hispanic breast cancer survivors and African-American cancer patients and survivors. In particular, Latino/Hispanic American breast cancer survivors frequently expressed information needs about reconstruction options, prosthetics, and clothing. On the other hand, Chinese cancer patients or survivors did not often discuss their information needs on body image and sexuality [42].

#### 3.3.1. Sexuality Information Needs

The evidence from the present study showed that sexual information needs were the most commonly reported subcategory within this category. The findings suggest that many breast cancer survivors experience problems such as sexual dysfunction, orgasm disorders, dyspareunia, sexual anxiety, and loss of libido [32, 34, 36, 38, 40].

### 3.4. Rehabilitation Information Needs

The combined evidence showed that rehabilitation information needs of breast cancer survivors existed in 8 subcategories, with the highest frequency being related to “self-care issues or home care during recovery/self-care practices” [21, 24]. Meanwhile, Riccetti et al. reported that rehabilitation information needs were concerned with nutrition to a great extent; they observed that this need was highly common among Chinese, Native American, Alaskan, and Middle Eastern patients with cancer or cancer survivors [42].

### 3.5. Prognosis Information Needs

The findings of the present review demonstrated that information needs regarding prognosis could be attributed to the following six subcategories: chances of cure, life span or survival rate, recurrence of cancer, risk of complications, survival statistics of treatment regimen, and awareness about possible prognosis.

Current evidence supports that the need for information on chances of cure is an important subcategory in this category [32]. Also, information needs about prognosis during diagnosis and treatment stages include overall survival rate and risk of recurrence [34]. In line with the present study, Riccetti et al. stated in their umbrella review that information needs about prognosis of cancer recurrence were often expressed by Chinese and Middle Eastern patients with cancer [42].

### 3.6. Cancer-Specific Information Needs

Based on this umbrella review, the information needs related to this category were identified in the following 5 subcategories with comparable frequency: etiology, diagnosis, clinical manifestations and symptom management, information about the nature of the disease, and information resources about diagnosis [31, 34, 40]. The findings also substantiated that the need for information on the etiology of breast cancer was one of the most important needs and that the main concern of breast cancer survivors about clinical manifestations was related to symptoms and fear of tumor recurrence [34]. Under similar circumstances, in a study on various types of cancer, Riccetti et al. confirmed that cancer-specific information needs were the most common needs reported during the stages of cancer diagnosis and treatment among most ethnic groups [42]. Furthermore, the participants' unmet information needs in the studies by Amiri et al. and Husson et al. emphasized information about the nature of the disease [6, 43].

#### 3.6.1. Where to Get Information about Specific Cancer Diagnosis or Treatment

According to the results of this review, due to the close relationship between diagnosis and treatment stages, the researchers integrated the information needs regarding the access to information resources in these 2 phases. Accordingly, this subcategory, which was included within the main categories of cancer specific and treatment by Rutten et al., was separately taken into account and included as another subcategory within the cancer-specific information needs category. The findings of the present review further confirmed that breast cancer survivors expressed a strong desire to receive health information even after diagnosis [34]. Information-seeking behaviors during diagnosis and treatment can emerge in the form of asking questions from healthcare providers, listening to stories and experiences of other people with cancer, searching for related resources, and even volunteering in research projects. According to the present findings, some resources were found as the most popular resources used by breast cancer survivors for information during diagnosis and treatment stages. These sources included medical records, libraries, audio/video resources, cancer organizations, other people's experiences (family, friends, coworkers, and peers/support teams), traditional media (e.g., radio & TV), attending educational programs, specialized applications, printed pamphlets, trustworthy websites on the web, and healthcare professionals (i.e., oncologists, nurses, physicians, and healthcare providers) [31, 34]. The findings reported by Riccetti et al. were in line with the present study in that both confirmed that Asian-European and Middle Eastern patients with cancer had a strong need for knowing “where to get information about treatment” [42].

### 3.7. Surveillance and Health Information Needs

The findings of the present review showed that information needs relating to surveillance and health could be placed in three subcategories, with the highest frequency being “maintaining psychological health.” In this regard, it was found that psychological health was one of the most common areas about which patients need information, which particularly applies to the fear of recurrence or cancer progression [40].

### 3.8. Medical System Information Needs

According to results, the information needs related to the medical system were expressed in three subcategories with the highest frequency being related to the following items.

#### 3.8.1. Interaction with Healthcare Providers

Evidence from the present review suggests that patients develop a basic need to obtain information from care providers [33, 38]. Similarly, in the study by Riccetti et al., information needs involving were common among patients with cancer from all ethnic backgrounds, except for Lati “healthcare system” no/Hispanic American patients [42]. Considering breast cancer survivors' attempts to obtain information from healthcare providers, the latter must feel committed and fully aware of updated clinical guidelines on cancer. In this regard, Niño de Guzmán et al. surprisingly observed that a large number of patients with breast cancer had not received information on the care recommended by clinical guidelines [44].

#### 3.8.2. Healthcare Systems/Quality of Medical Equipment and Supplies

The results of this umbrella review showed that the need for information about the healthcare system was among the most critical needs. For example, information regarding a better medical center, name of an oncologist, roles of different hospital staff in the department, information about logistic units or support departments within a medical facility, and even awareness about the quality of medical care-provision and high-tech equipment were highly necessary for breast cancer survivors [32, 39, 40]. Furthermore, factors such as old age, history of mastectomy, and comorbidities, as well as experiencing progressive stages of cancer directly influenced and accentuated this information need [39].

### 3.9. Coping Information Needs

The findings of this umbrella review reflect the information needs for coping with breast cancer in line with their emotional reactions as described in the following.

#### 3.9.1. Emotional Reaction or Emotional Support for Coping with Cancer

Based on our findings, the need for effective coping strategies is among the information needs of breast cancer survivors for dealing with stressful complications during their experience of the disease.

### 3.10. Interpersonal/Social Information Needs

The results of the present review revealed that information needs concerning interpersonal and social relationships were common in the effects on family, friends, and caregivers, subcategories [20, 24, 31, 34, 38]. Riccetti et al. confirmed the prevalence of information needs regarding the effects on family, friends, or caregivers, among Latin American/Hispanic cancer survivors as well as African American cancer patients and survivors [42].

### 3.11. Financial/Legal Information Needs

According to the findings of this review, some of the information needs of breast cancer survivors concerned in financial/legal information needs category were related to the costs of treatment, insurance coverage, and financial problems.

#### 3.11.1. Costs of Treatment, Insurance Coverage, or Other Financial Issues

Patients generally seek information about insurance coverage during diagnosis and treatment stages, as well as information about further financial support (e.g., unemployment and sources of financial assistance) during cancer survivorship [34, 40]. Riccetti et al., for instance, reported that Latino, Hispanic, and African American women with cancer often expressed information needs regarding financial difficulties, inability to support family expenditure, and their feelings of being humiliated due to financial needs. Indian-American and African-European patients with cancer also reported that they urgently needed information about insurance, financial support, and out-of-pocket payments, whereas Chinese patients with cancer rarely mentioned financial and legal information needs [42]. Notably, financial toxicity remains a major problem for breast cancer care recipients [45]. For instance, Thai breast cancer survivors became anxious when they found that insurance only covered certain costs of medical care and treatment. Patients not only in Thailand but also in many other countries expressed their need for wigs, transportation facilities, and life-sustaining needs; consequently, they were urged to request a wider range of insurance coverage [46]. It should be emphasized that patients who reported cancer-related financial problems or high treatment costs might eventually either halt or postpone prescribed medication and medical care as a result of increased financial obligations and accumulated debt [45].

## 4. Discussion

This umbrella review investigated 14 systematic reviews and classified the information needs in 11 main categories and 86 subcategories. The geographic distribution of needs in these studies reflects these information needs at a global level (Figure 2). The higher frequency of published articles in Asian countries could be attributed to the following two factors: the cultural diversity on this continent and the contextual nature of those needs. The increase in the number and changes in the main categories and subcategories of information needs in the present umbrella review relative to the study of Rutten et al. could be discussed in light of the time interval between the two studies and the fact that the present study focuses on the needs of women as a distinct social group.

The information needs identified in this review were as follows in the descending order of frequency: treatment related, supportive care, body image/sexuality, rehabilitation, prognosis, cancer specific, surveillance and health, medical system, interpersonal/social, coping, and financial/legal. In the following, some important results will be discussed and compared with other studies.

### 4.1. Treatment-Related Information Needs

With regards to treatment-related information needs, the long list of identified subcategories confirms the diversity of information needs regarding treatment issues among breast cancer survivors. Another significant finding of this umbrella review was that obtaining information about treatment plan and decisions is one of the fundamental needs among women undergoing chemotherapy and older women when they have to accept or reject treatment or make a choice of specific treatment methods [24, 38]. This challenge is apparently due to the presence of a heterogeneous spectrum of people in the population of older women who survive breast cancer. While some older patients continue to live an independent and otherwise healthy life, many of them often have comorbidities and cognitive and sensory impairments [34]. Therefore, the risk of mortality from causes other than breast cancer increases with age, and the adoption of different treatment approaches is associated with complications. In addition, older women have different priorities compared with younger women; the former may be less willing to prefer quality of life over prolonged survival. Meanwhile, our findings indicated that older adults might opt to either of the following three therapeutic decision-making styles: physician-based, patient-based, and shared decision-making [34].

In this umbrella review, we found that obtaining information about different diagnostic tests as well as the waiting time for results was another basic need of breast cancer survivors [34, 38]. The authorities in charge should be aware that responding to this need was among key performance indicators of the health system [47]. Indeed, the World Health Organization advises countries to make a diagnosis of the breast cancer within 60 days after the first visit and start treatment within 3 months after it [48]. Young women undergoing mastectomy or breast-conserving therapy have also expressed the need to know about the effects of clinical treatment on menopause, fertility, contraception, pregnancy during or after treatment, and breastfeeding [34, 38]. Although these patients may not have even considered having children before the diagnosis of cancer, the idea of possible infertility or menopause after treatment can be a stressful experience [49]. In this regard, Peate et al. highlighted multiple information needs among breast cancer survivors in relation to menstrual changes, possible infertility attitudes, as well as actual decisions about breastfeeding and contraception [50].

### 4.2. Supportive Care Needs

What has been identified through this umbrella review is that in the supportive care category, physical, social, spiritual, and features of information needs in breast cancer survivors appear to be the notable and challenging items. However, another umbrella review which was conducted on various cancers' unmet supportive care needs reported that psychological needs were at the center of attention, and this need has been reported as a common factor affecting different domains of supportive care needs. Therefore, cancer treatment must have a comprehensive approach and examine all aspects of a person's life [26].

### 4.3. Physical Needs

Elderly women with cancer encountered challenges such as lack of a personal transportation vehicle, long hours of waiting time in medical centers, multiple hospital visits and multicentral treatment, inability to do activities of daily living they used to do, lethargy, and feeling of frustration [21, 32, 33, 37, 40]. These treatment-related factors, which may not seem crucial, play a decisive role when patients are supposed to decide about their treatment. For example, radiotherapy and chemotherapy, which require going to multiple departments, can cause difficulty for patients lacking personal transportation vehicles. In addition, tolerating the waiting time in therapy sessions can be overwhelming for older women [37]. On the other hand, since old age is associated with loneliness, the onset of depression symptoms and reduced physical strength in patients with breast cancer [51], the impact of treatment on body functions, and independence can gain more importance than survival outcomes [52]. In this regard, Amiri et al. reported that breast cancer survivors who received chemotherapy or radiotherapy or both commonly needed information about managing physical symptoms such as pain, lymphedema, reduced ability to do daily activities, dyspepsia, and hair loss [6].

### 4.4. Social Needs

The findings of this review revealed that fulfilling the need for social support, especially by family members and health care providers, can reinforce the effect of self-care, assisting patients in accepting their condition and improving the distress associated with treatment [24, 32, 38]. In this regard, Ursavaş and Karayurt pointed out that since providing medical treatment procedures (such as surgery, chemotherapy, radiotherapy, and hormone therapy) for women with breast cancer was not adequate to overcome the disease, one should develop and implement interventions based on support groups so that these survivors could feel that there are other women with breast cancer under similar conditions and that they were not alone. Such supportive interventions help cancer survivors find the right resources for information while enjoying the benefits of peer support [53]. Likewise, Jablotschkin et al. reported that participation in peer-led self-help groups could be fruitful for patients with cancer, having advantages such as information support, shared experience and learning, helping others, and cultivating humor as a coping strategy [54].

### 4.5. Spiritual Support

Another significant finding in the context of supportive care needs was related to mental and spiritual needs. The need for mental energy may be considered as part of a support system. Friends' encouragement, shared experiences with other patients, and spiritual serenity derived from religious and soothing beliefs can help breast cancer survivors overcome their morbid condition with feelings of tranquillity, security, and complacency (36) in order to courageously face the uncertainty of the present and future situations [31]. Other studies have confirmed that spirituality is a significant resource when making decisions about diagnosis and treatment [55]. It is also a major coping strategy under stressful situations experienced by women with breast cancer, which is useful for coping with the disease and its treatment complications [56].

While spirituality creates positive effects, it should be noted that patients' religious and spiritual values may denote different concepts and occasionally induce emotional distress [57]. Therefore, healthcare providers and psychosocial consultants should explore emerging situations and take timely interventional steps to help women with breast cancer deal with negative aspects as well because these may affect their adjustment mechanisms during the first year after diagnosis [58]. In this umbrella review, we found that young women surviving breast cancer needed even more information about sexuality and body image, family, and treatment and that they had greater concerns about physical appearance and gender issues [38]. While certain changes such as hair loss may be of a temporary and short-term nature, they may exert a profound effect on how a woman can feel about herself [59]. In this regard, Thakur found that body image was one of the essential components of women's health and that healthcare providers should attempt both to prevent patients' mental and physical problems and stick to their treatment regimen [60]. Vegunta et al. observed that while sexuality issues constituted a common concern among breast cancer survivors, they were often overlooked in both clinical and research settings [61]. Therefore, the care of breast cancer survivors should include serious interventions to cater for sexuality disorders [62].

### 4.6. Information Needs and the Features of Information

According to the findings of our review, breast cancer survivors need to understand the features of information and methods of receiving information throughout different stages of treatment [31, 33, 34, 40]. In general, the supportive care needs are dependent on cultural context [33]. Because patients' information needs are likely to be affected by various factors such as gender, age, educational level, occupation, and cultural diversity, care providers must consider all these factors in providing information to cancer patients. For example, traditionally, healthcare workers directly provide health information to breast cancer survivors either through face-to-face conversation or through giving them handouts. Despite the validity of traditional routes of transferring information, they are often less effective during the early stages of cancer diagnosis because women may experience a rather perplexed state of mind at the beginning. In addition to the methods of information provision, characteristics of pieces of information are critical too. Appropriate and timely information provision can improve the level of coping with cancer through the reinforcement of the spirit of fighting against cancer and overcoming hopelessness. Meanwhile, inconsistent or inadequate information may contribute to the confusion of patients about disease prognosis. While overwhelming information in turn contributes to patients' anxiety, insufficient information or lack of trustworthy information can cause undue uncertainty about the treatment process. It should be expounded, however, that although many patients would like to receive information directly, gradually, and positively, others prefer alternative approaches and even not to receive explicit information [31].

### 4.7. Self-Care Issues or Home Care during Recovery/Self-Care Practices

The findings of the current review indicated that the most frequent information needs about rehabilitation in women undergoing chemotherapy concerned their empowerment in self-care practices at home during the recovery period [34]. Patients also prioritize receiving self-care instructions [32]. Nguyen observed that self-care strategies after discharge grew in popularity among breast cancer survivors and their family members as a means to help maintain quality of life and to reduce psychological distress [20]. It is worth noting that although self-care is among the most important principles of life and a successful treatment for patients with cancer, the first step and the most effective measure for self-care is the expansion of their health literacy [63]. Therefore, Deveci et al. recommended that healthcare professionals should educate patients with breast cancer about facts on self-care practices after breast cancer surgery [64].

### 4.8. Prognosis Information Needs

According to the results of the present study, although it is necessary for breast cancer survivors to know the risk of recurrence during the diagnosis, treatment, and survival stages, they also need information about the risks of complications and recurrence as well as the survival rates of different therapies to make treatment decisions [34]. In this regard, Kim et al. found that many breast cancer survivors barely knew about ductal carcinoma in situ and were likely to misunderstand the associated risks and prognosis; consequently, their anxiety level after treatment slightly differed from that of women with invasive breast cancer [65].

### 4.9. Surveillance and Health Information Needs

Our findings indicated that a third of patients with metastatic and recurrent breast cancer were at risk of experiencing varying degrees of depression. Higher rates of depression in this group show that cancer patients need effective psychological support not only at the time of diagnosis but also during the entire treatment period [35]. In this regard, Dinapoli et al. confirmed that breast cancer was the prelude to a stressful process for a patient who has to grapple with unknown challenges and difficult choices. Accepting the diagnosis, following the treatments, understanding the prognosis, and managing possible side effects and possible recurrence, as well as facing an uncertain future are major stressful steps that can result in mental instability, depression, or other mood disorders [66].

### 4.10. Coping Information Needs

Coping strategies often differ from individual to individual and are highly dependent on a patient's coping style, their experience of symptoms, need to control symptoms, and approach to challenges, as well as knowledge and beliefs, in addition to how chemotherapy affects their life. According to the combined evidence, three coping styles were identified in women with breast cancer undergoing chemotherapy as follows:

1. Emotion-focused coping strategies,
2. Problem-focused coping strategies,
3. Behavioral coping strategies.

Our findings support the stance that emotion-focused, problem-focused, and behavioral-coping strategies are effective tools for mitigating symptom-related distress and enabling patients to exert control over their lives. The purpose of coping strategies is to analyze and discover the causes of stressful events, seek relevant information and social support, eliminate or resolve problems, set goals, and modify personal expectations. Emotion-focused coping strategies are not intended to directly resolve problems; rather, they aim to modify one's responses such as crying, denial, acceptance, hopeful thinking, sense of humor, distraction, and avoidance. Behavioral coping strategies such as remaining hopeful, understanding and following self-care instructions, constant self-appraisal to ensure one's health, and attempting to maintain one's active role in life can improve the patient's quality of life and facilitate coping with adverse symptoms [24]. Riccetti et al. concluded that among Asian American and African American survivors of cancer, the most essential information needs were about “coping information,” especially “emotional reactions, emotional support, coping with cancer” (anxiety and depression) [42].

### 4.11. Effects on Family, Friends, and Caregivers

Based on the findings of this umbrella review, breast cancer survivors are in need of information on how to communicate about cancer with their family [31, 38], young children, and adolescents [34]. The results of a related systematic review indicated that mothers diagnosed with breast cancer encountered challenges in revealing their condition and related matters to their children. However, it was ultimately discovered that these mothers did disclose their illness and associated concerns to their offspring. This outcome might be attributed to the fact that it is challenging for mothers to maintain complete secrecy from their children who live with them [67].

Other studies have suggested that teenage girls experience more emotional distress compared to male counterparts due to heredity issues as well as fertility risks they might encounter at different periods of life like their mothers. After being diagnosed with breast cancer, teenage girls' mothers constantly feel distressed with the disease. Also, the treatment negatively affects teenage girls' mental health, learning, and daily life. Their mothers even developed worries about the impact of the disease on their daughters' marriage opportunities and economic prospects [68].

## 5. Conclusions

Evidence indicates that women diagnosed with breast cancer experience a multifaceted condition affecting a specific body organ that is of paramount importance to their femininity. Indeed, the diagnosis and treatment of breast cancer are likely to have a significant impact on their physical health and their psychological and social lives.

In the present umbrella review, the researchers identified key information needs of breast cancer survivors, including treatment information, supportive care, and body image/sexuality information needs that require immediate attention. Although the highest frequency of information needs was related to the three aforementioned items, it is evident that all identified areas require interventions to meet patients' information needs. Furthermore, from the geographical distribution of information needs around the world, it can be argued that breast cancer has become a global priority. This is due to a number of factors, including the transformation of breast cancer into a chronic disease, concerns over challenges related to information overload and the quality of available information, and the importance of the breast as the most important external identification of femininity.

This umbrella review of existing studies on identifying and documenting the information needs of breast cancer survivors reveals a substantial body of research in this field. It appears that researchers in this field have reached a point where they can transition from the exploratory stage to the operational stage with greater seriousness. Survivors are currently awaiting actions to address these needs from policymakers and healthcare providers. It is evident that countries that assume a pioneering role in this transition and operational stage and focus on breast cancer survivors' needs in providing health services become healthier, more dynamic, and most importantly, more productive societies. The advantages and positive consequences of this concentration will be experienced by the present generation and those to come.

## 6. Implications for Research

It is suggested that another umbrella review be conducted on the impact of interventions implemented so far in order to meet the information needs of breast cancer survivors and help identify the best practices in this field so that unnecessary parallel work is avoided, and practical measures are promptly adopted for similar patients in other regions.

## 7. Limitations

Umbrella reviews involve limitations as well. This type of study relies on information from articles that are covered in systematic reviews. As a result, an umbrella review cannot identify a phenomenon that has not been addressed in systematic reviews. The present research is not an exception. Therefore, our results should be interpreted with due attention to this limitation.

## Figures and Tables

**Table 1:** The search strategy of included databases and search engine.

Database/Date	Search strategy	# Results
PubMed/March 2024	(“Breast neoplasms”[MeSH terms] OR “breast cancer”[Title/Abstract] OR “breast neoplasm^*∗*^”[Title/Abstract] OR “breast tumor^*∗*^”[Title/Abstract] OR “malignant tumor of Breast”[Title/Abstract] OR “breast malignant tumor^*∗*^”[Title/Abstract] OR “cancer of the Breast”[Title/Abstract] OR “cancer of Breast”[Title/Abstract] OR “malignant neoplasm of Breast”[Title/Abstract] OR “mammary cancer^*∗*^”[Title/Abstract] OR “human mammary Carcinomas”[Title/Abstract] OR “human mammary neoplasm^*∗*^”[Title/Abstract] OR “breast Carcinoma”[Title/Abstract] OR “breast Carcinomas”[Title/Abstract]) AND (“health services needs and Demand”[MeSH terms] OR “informational need^*∗*^”[Title/Abstract] OR “information need^*∗*^”[Title/Abstract] OR “unmet need^*∗*^”[Title/Abstract] OR “supportive care need^*∗*^”[Title/Abstract] OR “health system need^*∗*^”[Title/Abstract] OR “psychological need^*∗*^”[Title/Abstract] OR “sexuality need^*∗*^”[Title/Abstract] OR “physical need^*∗*^”[Title/Abstract] OR “daily need^*∗*^”[Title/Abstract] OR “living need^*∗*^”[Title/Abstract] OR “patient care need^*∗*^”[Title/Abstract] OR “support need^*∗*^”[Title/Abstract] OR “health services need^*∗*^”[Title/Abstract]) AND (“women”[MeSH terms] OR “female”[Title/Abstract] OR “woman”[Title/Abstract]) AND (meta-analysis[Filter] OR review[Filter] OR systematic review[Filter])	34

Embase/March 2024	((“Breast cancer”: ti, ab, kw OR “breast neoplasm^*∗*^”: ti, ab, kw OR “breast tumor^*∗*^”: ti, ab, kw OR “malignant tumor of breast”: ti, ab, kw OR “breast malignant tumor^*∗*^”: ti, ab, kw OR “cancer of the breast”: ti, ab, kw OR “cancer of breast”: ti, ab, kw OR “malignant neoplasm of breast”: ti, ab, kw OR “mammary cancer^*∗*^”: ti, ab, kw OR “human mammary carcinomas”: ti, ab, kw OR “human mammary neoplasm^*∗*^”: ti, ab, kw OR “breast carcinoma”: ti, ab, kw OR “breast carcinomas”: ti, ab, kw OR “patients with breast cancer”: ti, ab, kw OR “breast cancer patients”: ti, ab, kw) AND (“health services needs”: ti, ab, kw AND demand: ti, ab, kw OR “informational need^*∗*^”: ti, ab, kw OR “information need^*∗*^”: ti, ab, kw OR “unmet need^*∗*^”: ti, ab, kw OR “supportive care need^*∗*^”: ti, ab, kw OR “health system need^*∗*^”: ti, ab, kw OR “psychological need^*∗*^”: ti, ab, kw OR “sexuality need^*∗*^”: ti, ab, kw OR “physical need^*∗*^”: ti, ab, kw OR “daily need^*∗*^”: ti, ab, kw OR “living need^*∗*^”: ti, ab, kw OR “patient care need^*∗*^”: ti, ab, kw OR “support need^*∗*^”: ti, ab, kw OR “health services need^*∗*^”: ti, ab, kw) AND (women: ti, ab, kw OR woman: ti, ab, kw OR female: ti, ab, kw)) AND “review”/it	81

Web of Science/March 2024	TS=((“breast cancer” OR “breast Neoplasm^*∗*^”OR “breast Tumor^*∗*^” OR “malignant tumor of breast” OR “breast malignant Tumor^*∗*^” OR “cancer of the breast” OR “cancer of breast” OR “malignant neoplasm of breast” OR “mammary Cancer^*∗*^” OR “human mammary carcinomas” OR “human mammary Neoplasm^*∗*^” OR “breast carcinoma” OR “breast carcinomas” OR “patients with breast cancer” OR “breast cancer patients”) AND (“health services needs and demand” OR “informational need^*∗*^” OR “information need^*∗*^” OR “unmet need^*∗*^” OR “supportive care need^*∗*^” OR “health system need^*∗*^” OR “psychological need^*∗*^” OR “sexuality need^*∗*^” OR “physical need^*∗*^” OR “daily need^*∗*^” OR “living need^*∗*^” OR “patient care Need^*∗*^” OR “support need^*∗*^” OR “health services Need^*∗*^”) AND (wom?n OR female)) and review article (document types)	187

Scopus/March 2024	(TITLE-ABS-KEY (“breast cancer” OR “breast Neoplasm^*∗*^” OR “breast Tumor^*∗*^” OR “malignant tumor of breast” OR “breast malignant Tumor^*∗*^” OR “cancer of the breast” OR “cancer of breast” OR “malignant neoplasm of breast” OR “mammary Cancer^*∗*^” OR “human mammary carcinomas” OR “human mammary Neoplasm^*∗*^” OR “breast carcinoma” OR “breast carcinomas” OR “patients with breast cancer” OR “breast cancer patients”) AND TITLE-ABS-KEY (“health services needs and demand” OR “neglected need” OR “informational need^*∗*^” OR “information need^*∗*^” OR “unmet need^*∗*^” OR “supportive care need^*∗*^” OR “health system need^*∗*^” OR “psychological need^*∗*^” OR “sexuality need^*∗*^” OR “physical need^*∗*^” OR “daily need^*∗*^” OR “living need^*∗*^” OR “patient care Need^*∗*^” OR “support need^*∗*^” OR “health services Need^*∗*^”) AND TITLE-ABS-KEY (wom?n OR female)) AND (LIMIT-TO (DOCTYPE, “re))	254

ProQuest/March 2024	Title (“breast cancer” OR “breast neoplasm” OR “breast tumor” OR “malignant tumor of breast” OR “breast malignant tumor”) AND abstract (“unmet information need” OR “informational need” OR “information need” OR “unmet need” OR “supportive care need” OR “health system need” OR “psychological need” OR “sexuality need” OR “physical need” OR “daily need^*∗*^” OR “living need” OR “patient care need” OR “support need” OR “health information need” OR “health services need”) AND abstract (woman OR women OR female) limits applied Databases: Publicly available content Database Narrowed by: Source type: Scholarly Journals	23

Cochrane Library/March 2024	#1 MeSH descriptor: [Women] explode all trees 1473 #2 female 959424 #3 #1 OR #2 959434 #4 (“health services needs and demand” OR (informational NEXT need^*∗*^) OR (information NEXT need^*∗*^) OR (unmet NEXT need^*∗*^) OR (supportive care NEXT need^*∗*^) OR (health system NEXT need^*∗*^) OR (psychological NEXT need^*∗*^) OR (sexuality NEXT need^*∗*^) OR (physical NEXT need^*∗*^) OR (daily NEXT need^*∗*^) OR (living NEXT need^*∗*^) OR (patient care NEXT Need^*∗*^) OR (support NEXT need^*∗*^) OR (health services NEXT Need^*∗*^)): ti, ab, kw (word variations have been searched) 6344 #5 MeSH descriptor: [Breast neoplasms] explode all trees 20048 #6 #3 AND #4 AND #5 107 Only select the cochran review: 2	2

Star is a truncation in strategy formulation, it is applied to enhance recall percentage in the retrieval process and searching for a word that could have multiple endings.

**Table 2:** The inclusion and exclusion criteria.

Inclusion criteria	Exclusion criteria
(i) Systematic reviews (ii) Articles focusing only on women breast cancer survivors (iii) Articles addressing the lack or inadequacy of health information needs of breast cancer survivors in at least one of the areas of sexual, psychological, and supportive care (including health and information system, emotional, physical, and daily, as well as spiritual and social needs)	(i) Articles addressing several other cancers (ii) Articles focusing on caregivers, family, friends, physicians, or service providers of breast cancer survivors (iii) Articles published in languages other than English (iv) Articles with unavailable full-text

**Table 3:** Assessment of methodological quality using JBI critical appraisal checklist for systematic reviews and research syntheses.

Studies	Questions											
	Q1	Q2	Q3	Q4	Q5	Q6	Q7	Q8	Q9	Q10	Q11	Quality rating
Khajoei et al. [30]	Y	Y	Y	Y	Y	Y	Y	NA	Y	Y	Y	10/11
Zou et al. [31]	Y	Y	Y	Y	Y	U	U	NA	Y	Y	Y	8/11
Nguyen [20]	Y	Y	Y	N	Y	Y	Y	NA	N	Y	Y	8/11
Liu et al. [24]	Y	Y	Y	Y	Y	Y	Y	Y	Y	Y	Y	11/11
Sheikhtaheri et al. [32]	Y	Y	Y	Y	N	N	Y	NA	Y	Y	Y	8/11
Armoogum et al. [21]	Y	Y	Y	Y	Y	Y	Y	Y	Y	Y	Y	11/11
Wabula et al. [33]	Y	Y	Y	Y	U	U	U	NA	Y	Y	Y	7/11
Lu et al. [34]	Y	Y	Y	Y	Y	U	Y	NA	Y	Y	Y	9/11
Karibayeva et al. [35]	Y	Y	Y	Y	U	U	Y	Y	Y	Y	Y	9/11
Masjoudi et al. [36]	Y	Y	Y	Y	Y	Y	Y	NA	Y	U	Y	9/11
Angarita et al. [37]	Y	Y	Y	N	Y	Y	Y	NA	NA	Y	U	7/11
Recio-Saucedo et al. [38]	Y	Y	Y	Y	Y	Y	Y	NA	Y	Y	Y	10/11
Lo-Fo-Wong et al. [39]	Y	Y	U	Y	U	N	U	NA	Y	Y	Y	6/11
Fiszer et al. [40]	Y	Y	Y	Y	Y	Y	Y	NA	Y	Y	Y	10/11

Y: yes; N: no; U: unclear; NA: not applicable; (Y) = 1.

**Table 4:** Characteristics of the included reviews based on JBI data extraction form for review for systematic reviews and research syntheses.

Study details			Search details					Analysis
Author/Year	Objectives	Participants	Sources searched	Range (years) of inch studies	Number of studies included	Types of studies included	Country of origin of included studies	Results/Findings
Zou/2022	To investigate the information needs of women; To explore the effects of information giving	537 women early-stage BC with no metastases or recurrence at the time of the studies	EBSCOhost CINAHL complete, MEDLINE, PsycARTICLES, PsycINFO, Web of Science, PubMed	2009–2019	6	Observational study, qualitative study, RCT	Italy, Turkey, Ireland, USA, Norway, Jordan	Prevalence of information needs; Effects of information giving are: (i) Appropriate and timely information (ii) Too much information (iii) Not enough/unavailable information

A. Angarita/2018	To identify, appraise, and synthesize the existing qualitative evidence on patient-reported factors influencing older women's decision to accept or decline breast cancer treatment	28 women who had already been counselled regarding breast cancer treatment and had either decided or completed their treatment	Medline, Embase, CINAHL, and PsycINFO	2000–2017	10	Retrospectively interviewed	Canada, UK (7), USA (2)	Finding patient-reported factor for accepting or rejecting treatment

Sheikhtaheri/2020	To evaluate the information needs of women with breast cancer	13185 women with breast cancer	PubMed, Scopus, Science Direct, and ProQuest	2010–2017	18	Cross-sectional descriptive, mixed methods (quantitative-qualitative)	Iran, Hong Kong, Lebanon, Turkey (2), Germany (4), Greece, Australia (2), USA (2), Jordanian, Canada, China, Italy	Most information needs were in the areas of diagnosis and treatment (first rank), daily activities (second rank), disease acceptance and self-image (third rank), personal and family life (fourth rank), and sexual health (fifth rank)

Hoai Thi Yen Nguyen/2022	To identify the postdischarge unmet supportive care needs of breast cancer patients; To collect related factors that affected those unmet needs	5262 women (postdischarge breast cancer)	PubMed, Wiley Online Library, and Science Direct	2011–2021	17	Cross-sectional studies (82.3%), one longitudinal study (5.9%), one retrospective (5.9%), and one prospective	Singapore (3), China (3), Japan (2), Belgium, Hong Kong and Germany, South Korea, Malaysia (2), UK, Netherlands, USA, Spain	Top ten unmet supportive care needs of postdischarge breast cancer patients (psychological + health system and information)

Armoogum/2020	To identify, review and synthesise qualitative research describing the experience of persistent pain in adult cancer survivors	52 women breast cancer survivors	CINAHL plus, Medline, PsycINFO, Embase, and Cochrane	2007–2019	4	Qualitative, longitudinal and prospective, explorative, phenomenological life world	Scandinavia (3), France	The main findings are as follows: (a) an interwoven relationship between experience of cancer and persistent pain, (b) lack of preparedness and support for persistent pain, (c) the physical impact of persistent pain, (d) employing coping strategies, (e) the emotional experience of persistent pain, and (f) conceptualisation of persistent pain

Masjoudi/2019	To review studies on women's sexual function using the female sexual function index (FSFI)	845 women with breast cancer were confirmed and completed the course of treatment (except for hormone therapy)	PubMed, Scopus, ProQuest, and Web of Science	2000–2017	5	Observational studies	Iran (5)	Based on the domains of FSFI, the lack of desire and lubrication dysfunction were the most common disorders while satisfaction with sexual life obtained the highest score

Lu/2020	To systematically identify, evaluate, and synthesize existing primary qualitative research on the information needs of breast cancer patients	3502	Web of Science, EBSCO, Scopus, ProQuest, PubMed, PsycINFO, the Cochrane Library, the Cumulative Index to Nursing and Allied Health Literature, Google Scholar	1996–2018	47	Empirical studies using qualitative or mixed research methods	United States (13), Australia (12), United Kingdom (7), Canada (10), Turkey (1), Japan (1), Iran (1), Poland (1), and Switzerland (1)	Three themes emerged: (1) incentives (physical abnormality, inquiry from others, subjective norm, and problems during appointments); (2) types of information needs (prevention, etiology, diagnosis, clinical manifestation, treatment, prognosis, impact and resumption of normal life, scientific research, and social assistance); (3) moderating variables (attitudes, health literacy, demographic characteristics, disease status, as well as political and cultural environment)

Deborah N. N/2015	To identify predictors of healthcare use among women with breast cancer; To review the evidence regarding the patient characteristics that were consistently associated with healthcare use of women with breast cancer	NA	PubMed, EMBASE, PsycINFO, CINAHL, and Cochrane Library databases. Also, backward and forward citation searches were performed	1994–2012	16	NA	NA	NA

Liping Liu/2021	To (a) explore the patient experience of chemotherapy, (b) identify patients' strategies to cope with the side effects and distress, and (c) explore the link between their experience and coping strategies	184 women with breast cancer who had received chemotherapy	PubMed, CINAHL, the Cochrane Library, PsycINFO, EBSCO host, Scopus, Embase, Web of Science, and reference lists of identified articles	1999–2017	12	Qualitative research	USA (2), UK (2), Turkey (2), Nigeria, China (2), Pakistan, Sweden, and Syria	Three synthesized findings were identified from 8 categories based on 91 original findings: (1) women living with chemotherapy experienced various stressful side effects, and their lives were changed. (2) Supportive care to address needs is essential to help women get through this difficult time. (3) They engaged in numerous types of coping strategies to deal with side effects and adapt to this difficult journey. Moreover, the link between experience of chemotherapy and coping strategies is based on the Lazarus' stress and coping theory

Chavie Fiszer/2014	To describes and analyzes the prevalence and predictors of the unmet supportive care needs of breast cancer (BC) patients and survivors and suggests paths for further research	6158 women diagnosed with BC	CINHAL, PubMed/Medline, and PsycInfo	2000–2013	23	Cross-sectional (19), longitudinal (5)	Japan (2), Australia (6), China (4), USA, (France, Switzerland), Turkey, Switzerland, Korea (2), (Germany, China), Taiwan, India, Germany (2)	The concerns of women diagnosed with BC clustered around psychological and information needs, with the top concern being “fear of the cancer returning.” Predictors of higher levels of needs included advanced disease stage, greater symptom burden, shorter time since diagnosis, higher levels of distress, and younger age. Prevalence differed between cultures with Asian women reporting greater information needs and lower psychological needs compared with western women

Irfan Wabula/2020	To summarize what is currently known about the unmet supportive care needs of breast cancer patients so that we are able to conduct the right interventions for the patients newly-diagnosed with breast cancer	5069 adult breast cancer patients	Scopus, ProQuest, Science Direct, and EBSCO	2014–2020	16	Cross-sectional (8), descriptive correlational study (3), quasiexperimental design, qualitative study (2), longitudinal study, retrospective study	Taiwan (2), Singapore, China (3), Malaysia (2), Thailand, Korea, Iran (4), Norway, Australia and African-American	The predictors of a higher level of need among the breast cancer patients are age, social support, and information. The most prevalent unmet needs among the women with breast cancer were found to be in the health system and related to information. The women with breast cancer who had more unmet needs in the physical and psychological domains were more likely to have a poor quality of life

Recio-Saucedo/2016	To determine information requirements and preferences of younger women diagnosed with early-stage breast cancer, facing a choice between mastectomy and BCS To determine young women's preferred information sources, including online and printed materials or decision aids, To identify the role of information in women's decision-making process	2574 young women diagnosed with early-stage breast cancer offered a choice between mastectomy and breast conservation surgery (BCS)	Medline, Embase, AMED; PsychInfo, PsychArticles, CINAHL, the Cochrane Controlled Trials Register for primary research, the database of abstracts of reviews of effects (DAREs) and the Cochrane database of Systematic Reviews (CDSR)	1995–2013	12	Cross-sectional (4), qualitative (6), longitudinal cohort, review,	Canada (1), UK (2), USA (5), Turkey (2), Hong Kong (2)	Findings indicate that young women prefer greater and more detailed information regarding treatment side effects, sexuality, and body image. Younger age of diagnosis leads to an increased risk perception of developing a second breast cancer. Young women's choices are influenced by factors associated with family and career

Karibayeva/2020	To systematically review current literature with data on the prevalence of depression symptoms in metastatic and recurrent breast cancer patients, examine the pooled mean prevalence of depression symptoms and potential sources of heterogeneity	1223 advanced breast cancer patients	PubMed, Web of Science, Scopus, Science Direct, Google Scholar, American Doctoral Dissertations and Open Grey databases	2007–2017	11	Cross-sectional (7), cohort (3), descriptive	Brazil, USA (5), China, Germany, Finland, Sweden, Czech	Patients with metastatic stage had a slightly higher prevalence of depression symptoms compared to recurrent breast cancer patients

Khajoei/2023	To identify supportive care needs from the point of view of breast cancer survivors	9730 breast cancer survivors	PubMed, Web of Science, and Scopus	1998–2021	40	Cross sectional (20), descriptive research Study (1), mixed methods study (1), retrospective study (1), correlational study (2), qualitative study (15)	USA (15), India (2), Korea (4), Singapore (2), Belgium (1), Australia (5), Malaysia (1), Taiwan (2), Denmark (1), China (1), Hong Kong (1), Japan (1), Spain (1), Canada (1), Netherlands (1), Mexico (1)	The most frequently mentioned supportive care needs of survivors were psychological/emotional needs (*N* = 32), health system/informational needs (*N* = 30), physical and daily activities (*N* = 19), and interpersonal/intimacy needs (*N* = 19)

**Table 5:** Summary of the findings.

Categories	Subcategories		References
Treatment-related information needs	Side effects of treatment/risks and benefits of treatment/management of side effects^*∗*^		[30, 32, 34, 38]
	Available treatments/treatment options/(factors that influence decision-making style to accept or decline treatment)^*∗∗*^		[24, 30–32, 34, 38]
	Treatment plan, treatment description, or logistical information		[34, 38]
	Other patients' experiences or treatment choices		
	Treatment success		
	Tests and procedures involved in treatment		[30, 34, 38]
	Physical effects of treatment^*∗*^		[21, 32, 37]
	Clinical trials/Scientific research^*∗∗∗∗*^		[34]
	Effects of missing treatment^*∗*^		[32, 34, 40]
	Treatment preparation^*∗*^		
	Prevention of recurrence^*∗*^		
	Treatment evaluation summaries^*∗*^		
	Alternative or complimentary treatments		
	Life-prolonging procedures^*∗*^		[24, 38]
	Impact on fertility, menopause, irregular menstruation, fertility contraception, pregnancy during or after treatment, and breast feeding^*∗*^		[30, 34, 38]
	Medication		[32]
	Progress during treatment		[32]
	Know necessary treatment^*∗*^		[31]
	Know anaesthetic and surgical procedures for before surgery^*∗*^		
	Follow-up after treatment^*∗*^		[32]
	Nutrition during treatment^*∗*^		[30, 32]
	Duration of the treatment^*∗*^		[32]
	Genetic counselling^*∗*^		[32, 38]
	Risks of persistent pain after cancer treatment^*∗*^		[21]
	Needing information about symptoms requiring a hospital visit and possible symptoms after hospital discharge^*∗*^		[30]

Supportive care needs	Informational needs and the features of information	Appropriate, accurate, and timely information^*∗*^	[24, 30, 31, 33, 40]
		Conflicting/inadequate information^*∗*^	
		Too much information^*∗*^	
		Not enough/unavailable information^*∗*^	
	Spiritual needs	Deal with unexpected outcomes^*∗*^	[24, 30, 31]
		Uncertainty resulting from limited information^*∗*^	
		Uncertainty related to the course of treatment^*∗*^	
		Uncertainty of living with cancer in everyday life^*∗*^	
	Social needs	Support groups^*∗∗∗∗*^	[24, 30, 32, 38]
		Support from other patients^*∗∗∗∗∗*^	
		Community counselling or support^*∗∗∗∗∗*^	
	Physical needs	Lack of energy/tiredness and pain^*∗*^	[21, 30, 32, 33, 37, 40]
		Not being able to do the things you used to do^*∗*^	
		Multiple hospital visits^*∗*^	
		Difficulties with transportation or take care of other family members^*∗*^	
		Appointments involve waiting^*∗*^	
	Emotional needs	Emotional experience of persistent pain^*∗*^	[21, 30, 32]
		Emotional side effects^*∗*^	
		Emotional responsibilities^*∗*^	

Body image/sexuality information needs	Sexuality information needs	The effect of breast cancer on body and sexual attractiveness and sexual activities^*∗*^	[30, 32-34, 36, 38, 40]
		Difficulties in being aroused/difficulties in reaching orgasm^*∗*^	
		Pain during intercourse^*∗*^	
		Information on sexual alteration^*∗*^	
		Anxiety about sex^*∗*^	
		Sexual well-being across stages of care^*∗*^	
		Sex aids and products^*∗∗*^	
		Sexual well-being for couples	
		Partner satisfaction, relationship communication, communication with partner^*∗*^	
		Increased sensitivity^*∗*^	
		Loss of sensation^*∗*^	
		How to talk about sex and intimacy^*∗*^	
	Understand exactly what was happening to their body and physical changes		[38]
	Physical appearance/physical attractiveness/skincare/prosthesis^*∗∗*^/needing information about loss of hair		[24, 30, 32, 34, 38]

Rehabilitation information needs	Self-care issues or home care during recovery/self-care practices^*∗∗*^		[20, 24, 32]
	Nutrition during recovery		[30, 34]
	Long-term side effects of cancer or treatment		[21]
	Being informed about things you can do to help yourself get well^*∗*^		[20, 30]
	Impact and resumption of normal life^*∗*^		[30, 32, 34]
	Services to support them with self-management of pain^*∗*^		[21]
	Exercise^*∗*^		[34]
	Vacations and travel^*∗*^		[32]

Prognosis information needs	Chance of cure		[30, 32, 34]
	Life span or survival rate		
	Recurrence of cancer/spread of cancer^*∗∗*^		
	Risk of complications^*∗*^		
	Survival statistics of treatment regimens^*∗*^		
	Know possible prognosis^*∗*^		[24]

Cancer-specific information needs	Etiology and course of disease		[20, 34]
	Specific diagnosis information		[31, 34]
	Symptoms of cancer/management of symptoms/clinical manifestation		[32, 34]
	Stages of disease		[32]
	Where to get information about specific cancer diagnosis/treatment^*∗∗∗*^		[31, 34]

Surveillance and health information needs	Prevention and early detection		[34]
	Eliminates their anxiety and overcome the disease/stress management/psychological needs^*∗∗*^		[30, 33–35, 40]
	Maintaining psychological health		[32-35, 40]

Medical system information needs	Interactions with healthcare providers		[24, 33, 38]
	Physical limitations during treatment^*∗*^		[37]
	Healthcare systems/quality of medical equipment and supplies^*∗∗∗*^		[32, 39, 40]

Coping information needs	Emotional reactions, emotional support, coping with cancer		[24]

Interpersonal/social information needs	Effect on family, friends, or caregivers		[20, 24, 30–32, 34, 38]

Financial/Legal information needs	Cost of treatment/insurance coverage/or other financial issues		[30, 34, 40]

^*∗*^ = new subcategories, ^*∗∗*^ = extended subcategories, ^*∗∗∗*^ = merged subcategories, ^*∗∗∗∗*^ = suggested subcategories, ^*∗∗∗∗∗*^ = moved subcategories.

## Data Availability

The data used to support the findings of this study are available on request from the corresponding author.
